# Supplementary material for: A quantitative exploration of symptoms in COVID-19 patients: an observational cohort study
Source: Int J Med Sci. 2021 Jan 1;18(4):1082–95. doi: 10.7150/ijms.53596 (PMC7807191; doi:10.7150/ijms.53596)
Supplement: Supplementary file 1 — Supplementary tables. [file ijmsv18p1082s1.pdf]

Supplementary Table 1 The correlation coefficients of inter-correlation between the 22 symptoms*

	A	B	C	D	E	F	G	H	I	J	K	L	M	N	O	P	Q	R	S	T	U	V
A	1.00	0.03	0.00	0.02	0.10	0.10	0.02	0.07	0.15	0.18	0.03	0.05	0.08	0.00	0.10	0.05	0.18	-0.06	0.07	-0.10	-0.07	0.08
B	0.03	1.00	0.49	-0.03	0.17	0.18	-0.06	0.10	0.13	-0.01	0.15	0.14	0.03	0.07	0.01	0.18	-0.06	0.10	-0.21	0.19	0.02	0.12
C	0.00	0.49	1.00	-0.02	0.22	0.31	0.09	0.18	0.18	-0.05	0.14	0.16	-0.05	0.03	0.00	0.30	-0.05	0.15	-0.14	0.13	0.03	-0.12
D	0.02	-0.03	-0.02	1.00	-0.01	0.06	0.00	-0.01	-0.08	0.14	0.06	0.25	-0.06	-0.01	0.03	0.12	0.06	0.30	0.00	0.05	0.00	-0.13
E	0.10	0.17	0.22	-0.01	1.00	0.48	0.09	-0.01	0.08	-0.06	0.70	0.13	-0.08	-0.15	-0.11	0.22	0.08	0.31	-0.10	0.01	-0.08	-0.08
F	0.10	0.18	0.31	0.06	0.48	1.00	-0.11	0.01	0.09	-0.11	0.45	0.11	0.00	0.01	0.05	0.39	0.14	0.30	-0.13	-0.10	0.09	-0.15
G	0.02	-0.06	0.09	0.00	0.09	-0.11	1.00	0.62	0.13	0.00	0.04	0.04	-0.07	-0.04	-0.06	-0.01	0.13	0.09	-0.03	0.13	0.17	0.01
H	0.07	0.10	0.18	-0.01	-0.01	0.01	0.62	1.00	0.03	-0.13	0.01	0.14	-0.10	-0.04	-0.05	0.03	0.07	0.00	-0.09	0.15	0.01	0.09

I	0.15	0.13	0.18	-0.08	0.08	0.09	0.13	0.03	1.00	-0.15	0.08	0.13	0.17	0.02	0.06	0.15	0.16	-0.09	0.18	-0.01	0.20	-0.11
J	0.18	-0.01	-0.05	0.14	-0.06	-0.11	0.00	-0.13	-0.15	1.00	0.00	0.15	0.03	0.05	-0.04	0.02	0.04	0.00	-0.02	-0.05	-0.11	0.11
K	0.03	0.15	0.14	0.06	0.70	0.45	0.04	0.01	0.08	0.00	1.00	0.11	-0.07	-0.14	-0.04	0.29	0.26	0.28	0.07	0.12	-0.01	0.00
L	0.05	0.14	0.16	0.25	0.13	0.11	0.04	0.14	0.13	0.15	0.11	1.00	0.02	-0.02	-0.01	0.11	0.05	0.08	-0.12	0.12	0.00	0.02
M	0.08	0.03	-0.05	-0.06	-0.08	0.00	-0.07	-0.10	0.17	0.03	-0.07	0.02	1.00	0.57	0.54	0.01	0.11	-0.08	0.13	0.21	0.04	-0.06
N	0.00	0.07	0.03	-0.01	-0.15	0.01	-0.04	-0.04	0.02	0.05	-0.14	-0.02	0.57	1.00	0.74	-0.02	-0.02	-0.10	0.09	0.14	-0.09	0.06
O	0.10	0.01	0.00	0.03	-0.11	0.05	-0.06	-0.05	0.06	-0.04	-0.04	-0.01	0.54	0.74	1.00	0.14	0.03	-0.10	0.08	0.13	-0.10	0.05
P	0.05	0.18	0.30	0.12	0.22	0.39	-0.01	0.03	0.15	0.02	0.29	0.11	0.01	-0.02	0.14	1.00	0.07	0.34	-0.08	0.13	-0.04	-0.09
Q	0.18	-0.06	-0.05	0.06	0.08	0.14	0.13	0.07	0.16	0.04	0.26	0.05	0.11	-0.02	0.03	0.07	1.00	0.06	-0.02	-0.05	0.14	0.11

R	-0.06	0.10	0.15	0.30	0.31	0.30	0.09	0.00	-0.09	0.00	0.28	0.08	-0.08	-0.10	-0.10	0.34	0.06	1.00	-0.07	0.02	0.07	-0.08
S	0.07	-0.21	-0.14	0.00	-0.10	-0.13	-0.03	-0.09	0.18	-0.02	0.07	-0.12	0.13	0.09	0.08	-0.08	-0.02	-0.07	1.00	0.04	0.07	-0.05
T	-0.10	0.19	0.13	0.05	0.01	-0.10	0.13	0.15	-0.01	-0.05	0.12	0.12	0.21	0.14	0.13	0.13	-0.05	0.02	0.04	1.00	0.03	0.01
U	-0.07	0.02	0.03	0.00	-0.08	0.09	0.17	0.01	0.20	-0.11	-0.01	0.00	0.04	-0.09	-0.10	-0.04	0.14	0.07	0.07	0.03	1.00	-0.08
V	0.08	0.12	-0.12	-0.13	-0.08	-0.15	0.01	0.09	-0.11	0.11	0.00	0.02	-0.06	0.06	0.05	-0.09	0.11	-0.08	-0.05	0.01	-0.08	1.00

*The 22 symptoms: (A) fever, (B) cough, (C) expectoration, (D) fatigue, (E) shortness of breath, (F) dyspnea, (G) nausea, (H) vomiting, (I) diarrhea, (J) chills, (K) chest distress, (L) poor appetite, (M) nasal obstruction, (N) runny nose, (O) myalgia, (P) palpitation, (Q) abdominal discomfort, (R) dizziness, (S) waist discomfort, (T) pharyngeal discomfort, (U) acid reflux, and (V) chest pain. Coefficient<0, negatively correlated. Coefficient>0, positively correlated. Coefficient ≥0.45, correlation was significant.

Supplementary Table 2 The results of the Lasso logistic regression to discriminate between the moderate type and the combined group of the severe and critical types

Model coefficient*	
Intercept	-2.96975438
Fever	.
Cough	.
Expectoration	0.17501574
Fatigue	.
Shortness of breath	1.87772614
Dyspnea	2.42226916
Nausea	.
Vomiting	-1.030009220
Diarrhea	0.22195853
Chills	.
Chest distress	.
Poor appetite	0.99170943
Nasal obstruction	.
Runny nose	.
Myalgia	.
Palpitation	.
Abdominal discomfort	.
Dizziness	.
Waist discomfort	-0.28647712
Pharyngeal discomfort	-0.02609929

Acid reflux	-0.55600809
Chest pain	.
Df = 9, dev.ratio = 0.5560217, nulldev = 177.4457, lambda = 0.03020582	
Penalty term lambda is the minimum lambda obtained from cv. glmnet function in R	

*, coefficient<0, the symptom was negatively correlated with the combined group of the severe and critical types. Coefficient>0, the symptom was positively correlated with the combined group of the severe and critical types.

Supplementary Table 3 The results of the Lasso logistic regression to discriminate between the severe type and the critical type

Model coefficient*	
Intercept	-0.7968486
Fever	.
Cough	.
Expectoration	.
Fatigue	.
Shortness of breath	.
Dyspnea	0.9464095
Nausea	.
Vomiting	.
Diarrhea	.
Chills	.
Chest distress	.
Poor appetite	.
Nasal obstruction	.
Runny nose	.
Myalgia	.
Palpitation	.
Abdominal discomfort	.
Dizziness	.
Waist discomfort	.
Pharyngeal discomfort	.
Acid reflux	.
Chest pain	.
Df = 1, dev.ratio = 0.1129977, nulldev = 55.35173, lambda = 0.1353652	

Penalty term lambda is the minimum lambda obtained from cv.glmnet function in R

*, coefficient<0, the symptom was negatively correlated with the critical type. Coefficient>0, the symptom was positively correlated with the critical type.
